# Leaky gut and intestinal mucosal injury among dogs with clinically subclassified atopic dermatitis

**DOI:** 10.29374/2527-2179.bjvm011725

**Published:** 2026-07-14

**Authors:** Kerem Ural, Songül Erdoğan, Hasan Erdoğan, Serdar Pasa, Tahir Özalp

**Affiliations:** 1 Department of Internal Medicine, Faculty of Veterinary Medicine, Aydın Adnan Menderes University, Aydın, Türkiye

**Keywords:** atopic dermatitis, dog, intestinal mucosae, intestinal permeability, leaky gut, dermatite atópica, cão, mucosas intestinais, permeabilidade intestinal, intestino permeável

## Abstract

We aimed to determine the relationships between intestinal mucosal injury (based on diamine oxidase [DAO] concentrations), physiological modulators of intercellular tight junctions, and leaky gut biomarkers (based on zonulin concentrations) in dogs with atopic dermatitis. We previously demonstrated that gut restoration alleviates canine atopic dermatitis (CaD) based on the interaction between the gut microbiome and dermatological diseases, which is referred to as the “gut-skin axis.” However, we observed the reverse in this study. This short-term, open-label, non-repeated study involved 30 owned dogs with CaD. Their CaD was clinically subclassified based on their Canine Atopic Dermatitis Extent and Severity Index version 4 (CADESI-04) scoring based on proposed benchmarks for mild (10), moderate (35), and severe (≥60) AD skin lesions]. Clinical interpretation and laboratory examination were composed of epidermal corneometric analytes (epidermal hydration and pH), CADESI-04 scores, and intestinal biomarkers. The DAO concentrations for the mild (≤10; Group I), moderate (≥11-35; Group II), and severe (≥60-180; Group III) groups were 2.1-6, 0.9-5.2, and 0.4-4.1 ng/mL, respectively. The data on the decline in DAO concentrations with disease progression were valuable (p = 0.031). The zonulin concentrations (ng/mL) for Groups I, II, and III were 14.67 ± 2.45, 13.40 ± 4.56, and 10.89 ± 6.49, respectively (p = 0.008). Their epidermal hydration levels for Groups I, II, and III (59.90 ± 17.77, 30.75 ± 14.59, and 17.58 ± 11.71, respectively) were statistically significant. However, their pH values for Groups I, II, and III (5.02 ± 0.64, 4.47 ± 0.35, and 4.66 ± 1.07, respectively) were not. This study highlights the exploration of the gut-brain-skin axis and leaky gut.

## Resumo

Levando em consideração a lesão da mucosa intestinal (através dos níveis de diamina oxidase [DAO]), o modulador fisiológico das junções intercelulares e o biomarcador do intestino permeável (detetado pelos níveis de zonulina) em cães com dermatite atópica, nosso objetivo foi detectar a relação entre eles. Além disso, como determinámos a interação entre o microbioma intestinal e as doenças dermatológicas, denotando o «eixo intestino-pele», demonstramos anteriormente que a restauração intestinal aliviou a dermatite atópica canina (CaD). Neste estudo, observámos o quadro inverso. Neste estudo original de curto prazo, aberto e não repetido, participaram 30 cães com CAD, subclassificados clinicamente [Índice de Extensão e Gravidade da Dermatite Atópica Canina versão 4 (CADESI-04), com pontuação baseada em referências propostas para lesões cutâneas leves (10), moderadas (35) e graves (≥60) de DA]. A interpretação clínica e o exame laboratorial foram compostos por análises corneométricas epidérmicas [ou seja, hidratação epidérmica e pH], (pontuações CADESI-04) e biomarcadores intestinais. No presente estudo, em relação à triagem CADESI-04, como leve ≤10 (Grupo I), moderada ≥11-35 (Grupo II) e grave ≥60-180 (Grupo III), os níveis de Dao foram determinados como 2,1-6, 0,9-5,2 e 0,4-4,1 ng/mL. Pareceram dados valiosos que, à medida que a doença progredia, os níveis de Dao diminuíam (p=0,031). Por outro lado, os níveis de zonulina (ng/mL) também diminuíram à medida que a doença progrediu: 14,67 ± 2,45, 13,40 ± 4,56 e 10,89 ± 6,49 (p = 0,008) para os grupos I, II e III, respetivamente. Em relação ao funcionamento da barreira epidérmica para os grupos I, II e III, respectivamente, os valores de hidratação epidérmica (59,90 ± 17,77, 30,75 ± 14,59 e 17,58 ± 11,71) foram considerados estatisticamente significativos, enquanto o pH para os grupos I, II e III, respectivamente (5,02 ± 0,64, 4,47 ± 0,35 e 4,66 ± 1,07) não apresentaram alterações estatísticas. Este é um estudo importante e pioneiro para explorar o eixo intestino-cérebro-pele e, consequentemente, o intestino permeável.

## Introduction

The gut-brain-skin axis has gained increasing attention in recent years as a multidirectional communication network linking the gastrointestinal system, central nervous system, and skin. Emerging evidence suggests that alterations in the gut microbiota and intestinal barrier function may contribute to the pathogenesis of dermatological diseases, including atopic dermatitis (Ural, 2023a). The concept of “leaky gut” has been proposed as a key mechanism underlying increased intestinal permeability and systemic inflammation (Camilleri, 2019; Quigley, 2016). This phenomenon has been associated with both gastrointestinal and extra-intestinal disorders (Nagpal & Yadav, 2017). In addition, dietary interventions, probiotics, and nutraceutical approaches have been suggested to modulate gut integrity and microbiota composition, potentially offering therapeutic benefits (Camilleri, 2019; Quigley, 2016).

It has also been linked to dietary interventions and probiotics and nutraceuticals, which have been suggested to improve gut integrity and serve as therapeutic strategies for various human diseases (Camilleri, 2019; Quigley, 2016). More studies have been conducted on administering beneficial live microorganisms to dogs. However, the understanding of the human gut microbiota in terms of the lining of the intestinal lumen, composition, and function has improved recently. This is largely attributable to new technologies that have revealed the genetic and metabolic profiles of the gut microbial strains as a new organ in the body, facilitating the development of a new promising therapeutic route for many disorders.

Probiotics have been suggested for the treatment of intestinal disorders in dogs, such as leaky gut and intestinal dysbiosis (Matei et al., 2021; McFarlin et al., 2017). McFarlin et al. (2017) reported that a rectal enema probiotic combination administered to patients with atopic dermatitis reduced circulating zonulin levels, indicating its effectiveness in alleviating chronic barrier disruption and restoring tight junctions. It also modified the microbiota (Ural et al., 2021a). In this study, the authors aimed to detect the probable relationships among leaky gut, tight junction abnormalities, and intestinal mucosal injury using relevant integumentary and intestinal biomarkers in dogs with clinically subclassified CAD.

## Materials and methods

### Subclassification of atopic dermatitis in dogs and relevant CADESI-04 scores

All cases were deemed eligible for enrollment, even if they met the diagnostic criteria for atopic dermatitis. The available criteria (similar to those used by Ural, 2023a) were based on clinical findings (Favrot et al., 2010; Griffin & DeBoer, 2001), exclusion of other relevant dermatoses based on the Favrot criteria (Favrot et al., 2010), and CADESI-04 scores based on the proposed benchmarks for mild (10), moderate (35), and severe AD (≥60) skin lesions (Olivry et al., 2014). In an attempt to exclude other relevant etiology; skin scraping, cutaneous cytology, dermatoscopy, epidermal corneometric analysis, serum biochemistry, and endocrine panel assessments were available (although the data are not presented herein). This study was approved by the Aydin Adnan Menderes University Local Ethical Committee on Animal Experiments-HADYEK (approval number: 64583101/2020/045; date: 9/7/2020). Epidermal corneometric analyses were performed using the Callegari Soft Plus Cornemetry Device at the Feline Dermatology Group facilities.

### Sandwich ELISA performance

The serum zonulin concentrations were determined using the Canine Zonulin (ZUN) ELISA Kit (MyBiosource). The kit had a sensitivity of 1.0 ng/mL and a detection range of 1.56-50 ng/mL. The standard concentrations (S6 to S1) provided by the manufacturer were 50, 25, 12.5, 6.25, 3.125, and 1.56 ng/ml. The intra-assay and inter-assay coefficients of variation were <15%. The sandwich ELISA was performed at the RDA Group facilities in Istanbul by an experienced specialist.

Intestinal mucosal injury was assessed using the Canine Diamine Oxidase ELISA Kit (MyBiosource). The relevant data were obtained from mybiosource website (https://www.mybiosource.com/dao-canine-elisa-its/diamine-oxidase/739902). The kits were purchased from RDA Group (Istanbul). The assay had a sensitivity of 1.0 ng/mL, and the specificity was as follows: spike recovery: 92-101%; linearity 1:8 range: 994-109%. The assay type was quantitative competitive and was performed using an ELISA device available at the RDA Group (Istanbul). In relation to the sensitivity, the detectable range was 0.312-20 ng/mL.

Prior to the RDA Group analysis in Istanbul. For both assays preparation and storage conditions composed of 2-8 °C. The Kruskal-Wallis one-way ANOVA test, a rank-based non-parametric method, was used. Statistical significance was set at p < 0.01.

Statistical analyses were conducted to evaluate the differences in epidermal hydration, pH, DAO concentrations, and zonulin concentrations among the three groups. The data were analyzed using R Studio with the “dplyr”, “ggplot2”, and “stats” packages. Statistical significance was set at p < 0.05. Descriptive statistics, including the mean and standard deviation, were calculated for each parameter within each group. One-way ANOVA was used to assess the differences between the group means, and Tukey’s HSD test was used for pairwise comparisons. Box plots showing the distributions of the parameters across the groups and individual data points were generated using R Studio.

## Results

**Figure 1:**
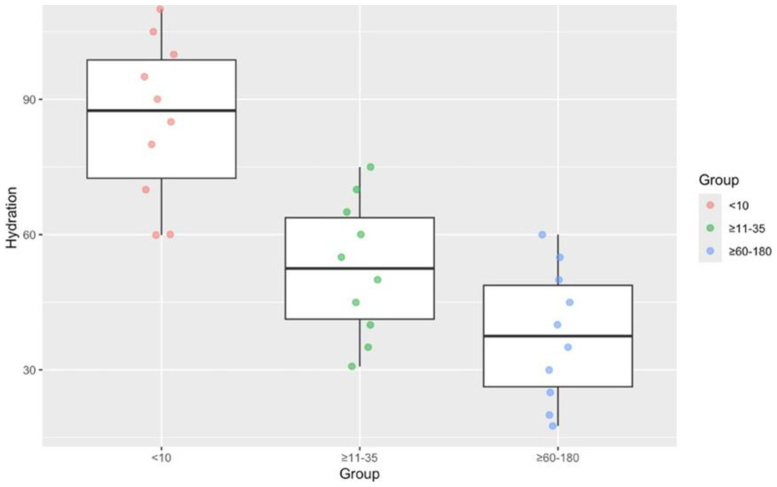
Boxplot analysis of epidermal hydration among 3 groups.

**Figure 2:**
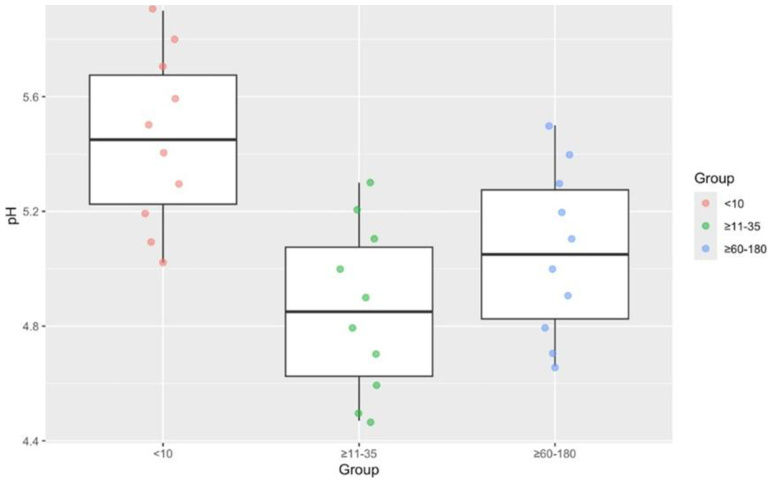
Boxplot analysis of epidermal pH among 3 groups.

**Figure 3:**
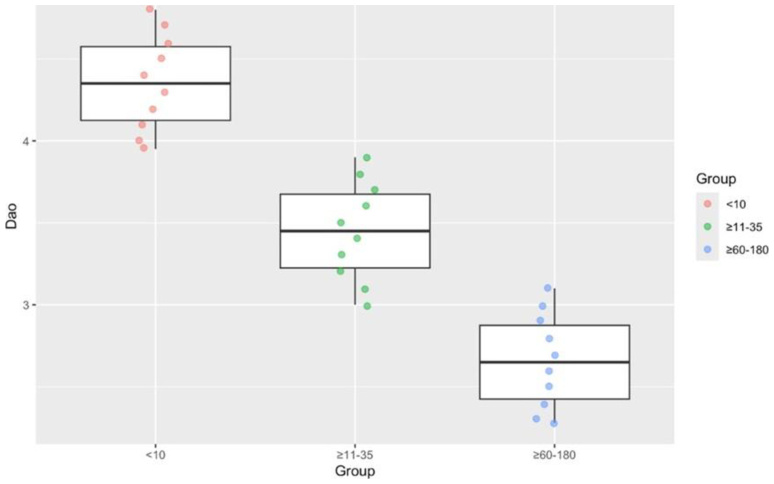
Boxplot analysis of Dao values (ng/mL) among 3 groups.

**Figure 4:**
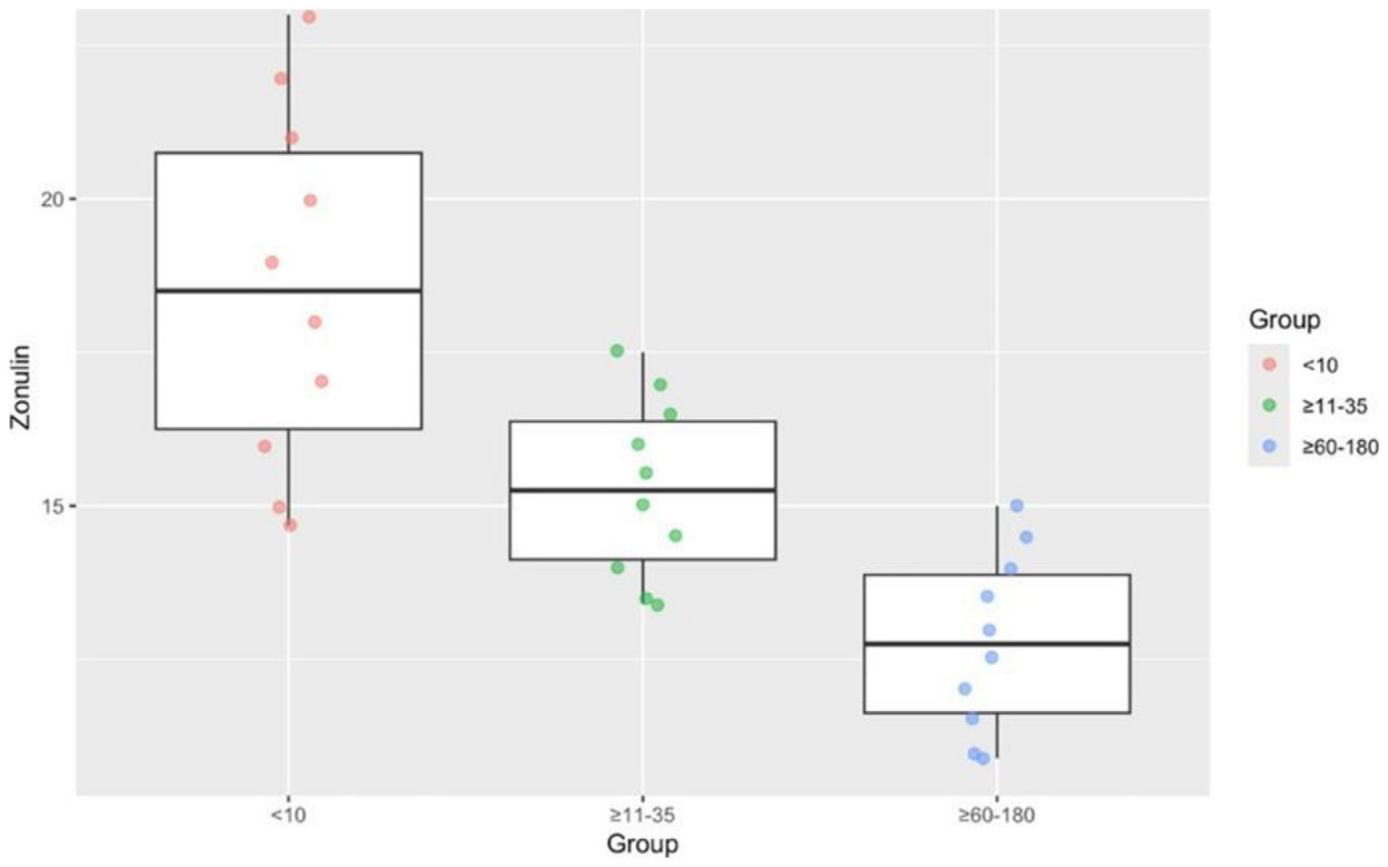
Boxplot analysis of zonulin values (ng/mL) among 3 groups.

The individual data and min-max results (Table 1) and statistical interpretation (Table 2) are provided sequentially as an open resource. The data for the three groups based on CADESI-04 triage (Group I, ≤10; Group II, 11-35; and Group III, 60-180) were significant and available. Boxplot analyses revealed significant changes, even at the set significance level for the p-values (Figures 1 to 4).

**Table 1:** Biomarkers and CADESI-4 scores showed min-max ranges in comparison to group individuals as pure material (data).

		**CADESI-04 triage**		
		**≤10**	**≥11-35**	**≥60-180**
Epidermal corneometry	Hydration	27-85	7-56	0-34
	pH	4.1-6.0	3.9-5.1	3.2-6.4
Dao concentrations (ng/mL)		2.1-6	0.9-5.2	0.4-4.1
Zonulin levels (mg/dl)		11-19	06-20	03-20

**Table 2:** Statistical evaluation of whole data.

**Parameter**	**Group 1**	**Group 2**	**Group 3**	**p-value**
**Hydration**	59.90 ± 17.77^a^	30.75 ± 14.59^b^	17.58 ± 11.71^c^	0.001
**pH**	5.02 ± 0.64	4.47 ± 0.35	4.66 ± 1.07	0.117
**Dao Concentrations** (ng/mL)	3.95 ± 1.30^a^	3.00 ± 1.38^b^	2.28 ± 1.13^c^	0.031
**Zonulin Levels** (ng/dL)	14.67 ± 2.45^a^	13.40 ± 4.56^b^	10.89 ± 6.49^c^	0.008

a,b,c Different letters in same row are statistically significant (p<0.001).

All authors declare that due to ethical guidelines and and animal welfare, all dogs enrolled were subjected to treatment with Pet Clinique Metabolique Tablets^®^ (Pet Clinique Veterinary Exclusive Series, Kepez, Antalya, TÜRKİYE), Pet Clinique Symbiotique Capsule^®^ (Pet Clinique Veterinary Exclusive Series, Kepez, Antalya, TÜRKİYE), Pet Clinique Easy Digestive Sol.^®^ (Pet Clinique Veterinary Exclusive Series, Kepez, Antalya, TÜRKİYE) and Celery Selection Liquid Sol.^®^ (Larek Tarım Ankara, TÜRKİYE), following the end of treatment. None of the dogs were remained untreated.

## Discussion

### Dedicated to detecting enemies

Could we suggest/speculate that our enemies [at the intestinal level of dogs with atopic dermatitis], the enemy [increased levels of Dao] was inside, not outside? This issue must be addressed at its source. DAO has been identified as the pivotal extracellular enzyme responsible for catabolizing histamine within the gastrointestinal canal (Maintz et al., 2011; Manzotti et al., 2016; Mušič et al., 2013; Sattler & Lorenz, 1990). It is, therefore, reasonable to conclude that this enzyme is capable of changing the approaches to managing food allergy and atopic dermatitis. The DAO concentrations for the mild (≤10), moderate (≥11-35), and severe (≥60-180) groups CADESI-04 traige were 2.1-6, 0.9-5.2, and 0.4-4.1 ng/mL, respectively. The data indicate that the DAO concentrations decreased as the disease progressed (Figure 3). This issue would thus be focused and discussed in detail.

If intestinal mucosal injury is the enemy [Dao decrease], should thus be anti-histamine rich foods? It is difficult to exclude/remove histamine rich foods

Ural (2023a) investigated intestinal integrity among dogs with mild (10), moderate (35), and severe (60) skin lesions based on their CADESI-04 scores. At that study very similarly and as our reference methodology commercially available Canine Diamine Oxidase ELISA Kit were used. Through Quantitative Competitive ELISA, DAO concentrations (ng/mL) for the groups were 5.28 ± 1.19, 2.594 ± 0.76, and 1.28 ± 0.22, respectively. The difference between the first and third groups was statistically significant. The author suggested that the serum DAO concentrations decreased with disease activity in canine atopic dermatitis. In contrast, the serum DAO concentrations were 3.95 ± 1.30, 3.00 ± 1.38, and 2.28 ± 1.13 in this study (p = 0.031) (Tables 1 and 2 and Figure 3). The plasma DAO concentrations reflect mucosal injury and plasma concentrations could reflect the integrity of the intestinal mucosa (Luk et al., 1981; Mennigen et al., 1987; Ural, 2023a; Zhou et al., 2006). Therefore, the dogs in the present study had severe intestinal mucosal injury, as CAD progressed. DAO activity reduces following several types of intestinal mucosal injury (Mennigen et al., 1987; Ural, 2023a; Zhou et al., 2006). We observed similar results in the present study. From this view, should we delve into further analyses to determine whether DAO or the histamine-rich foods is the enemy?

The importance of biogenic amines in pet foods and histamine intolerance has been reported (Blonz & Olcott, 1978; Craig, 2019; Verlinden et al., 2006). They are not limited to quality demands. Vasoactive amines (histamine and tyramine) could be capable of influencing cardiovascular system (European Food Safety Authority, 2011). Histamine has been described as a hazard (European Pet Food Industry Federation, 2017) that requires consideration. All amines have been indicated as freshness indicators (Midwest Laboratories data). The biogenic amine index for fish (Mietz & Karmas, 1978) does not include tyramine, which is abundant in meat (Ruiz-Capillas & Herrero, 2019). The biogenic amine and polyamine content of the inner tissues could be higher than those in the muscles (Fuchs et al., 2009) and processed commercial pet foods. The biogenic amine indices of several pet foods consumed by the 30 dogs involved in this study were unknown at the time of writing this manuscript. However, the histamine tolerance and biological mine indexes of most of the dogs may have been assigned based on the premise that they were fed fish or red meat as the primary protein source. This must be addressed by commercial companies. Decreased DAO concentrations should be related to this issue, at least for the dogs involved in this study.

### Zonulin, an enterotoxin, is a friend or foe?

In the present study, zonulin concentrations, which are indicative of leaky gut (Fasano, 2020; Hoshiko et al., 2021), were determined for the dogs with CAD based on their clinical subclassifications. In the present study, zonulin levels (ng/mL) were 14.67 ± 2.45, 13.40 ± 4.56, and 10.89 ± 6.49 for groups I, II, and III, respectively (p = 0.008). This might be a clue for the relationship between leaky skin (CAD) and leaky gut. The evidence for the gut-brain-skin axis and its quality should be improved based on clinical research, as reported herein. This section may attract criticism for repeatedly selecting zonulin in our studies, but the issue should be addressed. The permeability of the intestinal epithelium to small materials depends on the modification of intercellular tight junctions. The zonula occludens (ZO-1) toxin (Zot/zonulin) has been considered as an enterotoxin secreted by intestinal epithelial cells following dietary or microbiota induction. It regulates tight junctions and intestinal barrier function (Campisi et al., 2016; Tajik et al., 2020). However, an altered intestinal barrier against pathogenic niches allows pro-inflammatory cytokines to leak into the intestinal system and blood. Zonulin reduces the expression of tight junction proteins, induces a T-cell-mediated mucosal inflammatory response, and alters the migration of immune cells between the intestine and blood (Dalton et al., 2014). Zonulin evokes the dismantling of tight junctions in the duodenum and other parts of the small intestine, resulting in leaky gut (Dalton et al., 2014). Several studies have reported the association of serum zonulin concentrations with increased intestinal permeability and their influence on immunological, neural, and endocrine pathways in patients with leaky gut (Delaney et al., 2019).

## Conclusion

The studies and presentations on the gut-skin-brain axis (Alıç Ural, 2022a, 2022b, 2022c, 2023, 2025a, 2025b, 2025c; Alıç Ural et al., 2021a, 2021b; Alıç Ural & Ural, 2023; Ural, 2020a, 2020b, 2023a, 2024; Ural et al., 2017, 2018, 2020a, 2020b, 2021c, 2021d, 2023) should be acknowledged. However, further studies are warranted. This study highlghts the association of the gut-brain-skin axis and intestinal and dermatological biomarkers. The data indicated that the trilogy of worsening leaky gut, physiological modulation of intercellular tight junctions, and intestinal mucosal injury contributes to the progression of CaD in dogs with clinically subclassified atopic dermatitis.
